# Contasure-needleless single incision slings versus transobturator slings (TOT/TVT-O) for female patients with stress urinary incontinence: a systematic review and meta-analysis

**DOI:** 10.1186/s12894-020-00622-5

**Published:** 2020-05-06

**Authors:** Zhenkai Luo, Binbin Jiao, Hang Zhao, Hailong Liu, Shicong Lai, Guan Zhang

**Affiliations:** 1 Peking University China-Japan Friendship School of Clinical Medicine, Beijing, China; 2 Department of Urology, China-Japan Friendship Hospital, Yinghuadong Road, Beijing, 100029 Chaoyang District China; 3 Graduate School of Peking Union Medical College, China-Japan Friendship Institute of Clinical Medicine, Beijing, China; 4 Graduate School of Peking Union Medical College and Chinese Academy of Medical Sciences, Beijing, China; 5 Department of Urology, Beijing Hospital, National Center of Gerontology, Beijing, China

**Keywords:** Female stress urinary incontinence, Mini-slings, Needleless, Transobturator sling, Zhenkai Luo and Binbin Jiao are co-first authors.

## Abstract

**Background:**

To assess the current evidence on the effectiveness and safety of Contasure-Needleless (C-NDL) versus transobturator slings (TOT/TVT-O) in the management of female stress urinary incontinence (SUI).

**Methods:**

A comprehensive literature review of articles that investigated the efficacy and safety of C-NDL and TOT/TVT-O was performed based on studies published before June 2019 and retrieved from PubMed, Embase, CNKI and the Cochrane Library. Two reviewers searched the literature, independently extracted the data and evaluated the quality of the data according to the inclusion and exclusion criteria. A meta-analysis was performed by using Review Manager 5.3 software.

**Results:**

Seven studies with 1188 SUI female patients without intrinsic sphincter deficiency (ISD) or mixed urinary incontinence were included. Our meta-analysis showed that the clinical efficacy of C-NDL is statistically non-inferior to that of TOT / TVT-O in terms of subjective cure rate [OR = 0.77, 95% confidence interval (CI) (0.53 to 1.10), *p* = 0.15] and objective cure rate [OR = 0.78, 95% CI (0.53 to 1.13), *p* = 0.19]. In addition, operating times were statistically shorter with C-NDL compared to TOT / TVT-O [mean difference (MD) = − 7.38, 95% CI (− 10.73 to − 4.04), *p* < 0.0001]. In terms of the postoperative visual analogue scale (VAS) and the incidence of postoperative pain, C-NDL has a greater advantage [MD = − 1.71, 95% CI (− 2.91 to − 0.50), *p* = 0.005]; [OR = 0.21, 95% CI (0.05 to 0.96), *p* = 0.04]. Complication rates were statistically similar between the groups, except for groin pain which was higher in TOT / TVT-O.

**Conclusion:**

Our data suggest that C-NDL slings have similar short-term efficacy as TOT/TVT-O in curing SUI patients. Compared with TOT/TVT-O, C-NDL is associated with a shorter operative time, and the incidence of postoperative pain is decreased. Nevertheless, these findings should be further confirmed through large-volume, well-designed prospective randomized controlled trials (RCTs) with long-term follow-up.

## Background

Stress urinary incontinence (SUI) is a common disease among middle-aged and elderly women. It is a widespread, global disease that affects 50% of women [1]. Symptoms of SUI are manifested as the involuntary flow of urine from the urethra when the patient coughs, sneezes or runs, all of which elevate abdominal pressure [2]. It is generally recognized that urethral closure pressure is the key factor maintaining continence [3]. When exertion raises the intra-abdominal pressure, an insufficient pressure of urethral closure will cause leakage. The lack of urethral closure pressure is associated with anatomic changes in the bladder and urethra [4, 5]. Surgical treatment is now prioritized because of its efficacy when conservative therapy fails [6]. The purpose of surgical treatment is to reconstruct the anatomy and function through surgery and to restore normal urine control. However, for patients with intrinsic sphincter deficiency (ISD) or urgency urinary incontinence, a high failure rate of anti-incontinence surgeries has been reported [7, 8]. Surgical treatment of SUI has been developed for more than a century, and up to 200 surgical procedures are documented in the existing literature. The Burch retropubic urethropexy or Marshall-Marchetti-Krantz (MMK) procedure through the retropubic routine was the gold-standard surgical treatment of SUI before the introduction of MUS [9]. Recently, the frequency of similar surgical approaches has decreased significantly. DeLancey proposed the hammock hypothesis in 1994 [10], which provided theoretical support for subsequent minimally invasive surgery, bringing revolutionary changes to surgery. Since the introduction of tension-free vaginal tape (TVT), similar surgical approaches have made great progress. At present, standard midurethral slings (SMUS) have become the first-line surgical treatment [6]. Among them, the transobturator sling (TOT/TVT-O) is widely used because of its high cure rate and lower number of complications [11–13]. However, persistent groin and thigh pain after surgery is the main complication affecting patient satisfaction [14].

Single incision mini-slings (SIMSs) were introduced in 2006 to make use of a shorter sling and a single vaginal incision [15]. The short- and medium-term efficacy of SIMS are still controversial due to its recent introduction. Currently, as a new category of SIMS, Contasure-Needleless (C-NDL) slings have a non-inferior cure rate and fewer complications than transobturator slings [16, 17]. To the best of our knowledge, there are no systematic reviews and meta-analyses of C-NDL slings. To better evaluate clinical efficacy and safety, we systematically assessed data about C-NDL and transobturator slings (TOT/TVT-O) to provide a reference for the surgical choice of SUI.

## Methods

### Search strategy

A comprehensive literature search was performed based on PubMed, Embase, CNKI and the Cochrane Library before June 2019. The following key words were used: “single-incision mini-sling”, “Contasure-Needleless,” “Needleless,” “transobturator slings,” “TVT-O,” “TOT,” and “stress urinary incontinence”. We defined no language restrictions. Additionally, manual searches of the references and citation lists of all relevant reviews were performed. The literature selection was performed following the search strategy promoted by the Preferred Reporting Items for Systematic Reviews and Meta-analysis (PRISMA) guidelines.

### Inclusion and exclusion criteria

The included studies met the following criteria: (1) the study type was randomized controlled trials (RCTs) or case–control trials (CCTs); (2) the study compared the efficacy and safety of C-NDL with TOT/TVT-O; (3) participants were females and diagnosed with SUI; (4) there was no statistically significant difference in the basic characteristics of the participants; and (5) the measurement outcomes included cure rate, surgery-related data, and postoperative complications.

Studies were excluded based on the following criteria: (1) no data were available for meta-analysis; (2) the study was not an original research study (i.e., it was a conference article, letter, comment, or review); (3) the follow-up time was too short, that is, less than 1 year; (4) the experimental group included other SIMSs; and. (5) patients were diagnosed with ISD or mixed urinary incontinence.

### Data extraction and quality assessment

The literature selection was completed according to the inclusion and exclusion criteria. Two reviewers (Z. L and B. J) independently extracted data and appraised both quality and content. The following items were extracted from each available study: first author, year of publication, country, study design, intervention, sample size, follow-up data, definition of subjective cure, definition of objective cure, relative outcome (including subjective cure rate, objective cure rate, operative time, hospitalization time, blood loss, visual analogue scale) and overall complications. All authors engaged in a discussion to resolve relative disagreements about eligibility and the senior reviewer (G. Z) made the final decision after discussion.

We evaluated the level of evidence (LE) for each selected article based on the criteria recommended by the Oxford-associated evidence-based medicine centre [18]. For methodological quality assessment, the Jadad scale [19] was used to assess the quality of RCTs. Studies with scores of 3–5 were defined as high-quality, and studies of scores of 0–2 were defined as low-quality. For CCTs, we used the Newcastle–Ottawa scale [20] to evaluate quality. We defined the studies with scores of 7–9 as high-quality, while those with scores of 0–6 were low-quality. The quality of the studies did not influence the decision to pool studies in the meta-analysis.

### Statistical analysis

All meta-analyses were performed using Review Manager 5.3 software. The mean difference (MD) or standardized mean difference (SMD) was used to evaluate continuous outcomes. For studies that expressed continuous data as medians and range values, we chose the statistical formula demonstrated by Luo and Wan et al. [21, 22] to count the means and standard deviations. The results are expressed as the risk ratio (RR) or odds ratio (OR) with a 95% confidence interval (CI) for dichotomous variables. The χ2 and I2 tests (I2 > 50% was regarded as substantial heterogeneity) were used to assess the heterogeneity of the study data. If heterogeneity was considered to be low, fixed-effects models were used for the meta-analyses. Otherwise, a random effects model was used to reduce the effect of statistical heterogeneity. The pooled effects were determined by the z test, and a *p* value < 0.05 was considered statistically significant. For several comparisons, sensitivity analyses were used. The results of the meta-analysis are expressed using forest graphs.

## Results

### Characteristics of the selected studies

**Fig. 1:**
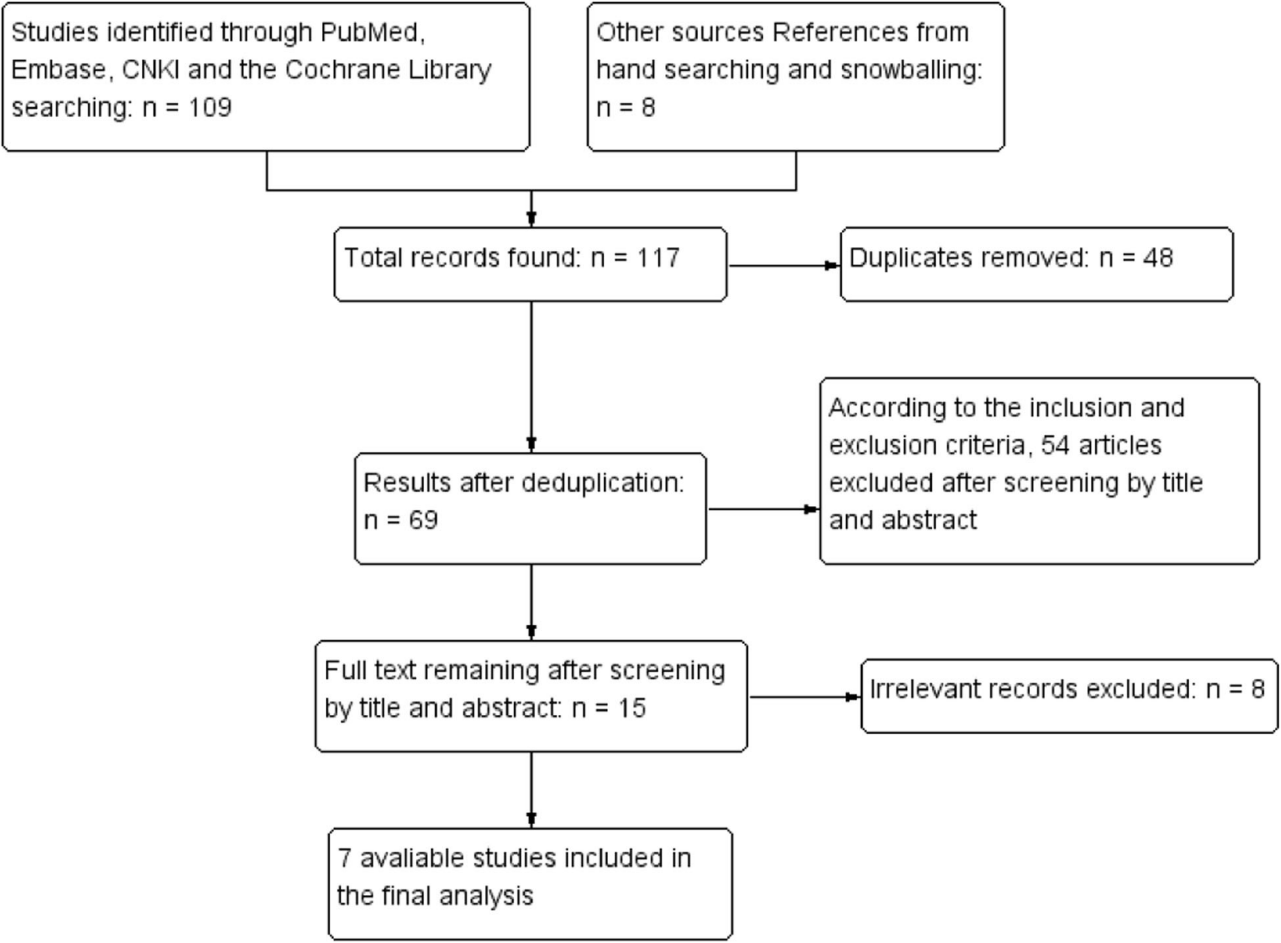
PRISMA flowchart

After the searches and screens, seven articles [17, 23–28] were ultimately included. The selection or exclusion of literature at each stage is presented in a flowchart (Fig. 1). A total of 603 cases of C-NDL and 585 cases of TOT/TVT-O were selected in this meta-analysis, including five RCTs and two CCTs. The baseline characteristics and quality assessment of the included studies are shown in a table (Table 1). According to the scoring criteria, these studies are defined as high-quality.

**Table 1:** Summary of comparative studies included in meta-analysis

Study	Country	Study period	Study design	LE	Intervention		Sample size		Follow-up	Study quality	Definition of subjective cure	Definition of objective cure
					Trial	Control	Trial	Control				
Dogan 2018 [23]	Turkey	2014–2016	RCT	2a	C-NDL	TOT	89	89	2 y	4^b^	If the response to the (ICIQ-SF) question 5 “When does urine leak?” was “never/urine does not leak”	Absence of SUI and negative CST
Fernandez 2017 [24]	Spain	2010–2014	RCT	2a	C-NDL	TVT-O	89	98	1 y	4^b^	Very satisfied/satisfied in questionnaire and postsurgical SSI is 0	Negative CST
Franco 2014[17]	Spain	2006–2010	CCT	2b	C-NDL	TVT-O	131	108	5 y	9^a^	Very satisfied/satisfied in questionnaire and postsurgical SSI is 0	Negative CST
Gaber 2016 [25]	UK	2014–2015	RCT	2a	C-NDL	TVT-O	70	70	1 y	4^b^	Very much improved/much improved on PGI-I	Negative CST with full bladder
Lv 2017[26]	China	2014–2015	RCT	2a	C-NDL	TOT	78	86	1 y	3^b^	PGI-I	Not mention
Tardiu 2011[27]	Spain	2006–2009	CCT	2b	C-NDL	TVT-O	72	60	1 y	9^a^	Very satisfied/satisfied in questionnaire and postsurgical SSI is 0	Negative CST
Xu 2017 [28]	China	2014–2016	RCT	2a	C-NDL	TVT-O	74	74	1y	3^b^	Postsurgical SSI is 0	Absence of SUI and negative CST

*RCT* Randomized controlled trial, *CCT* Case–control trials, *LE* Level of evidence, *C-NDL* Contasure-Needleless single incision slings, *TVT-O* Tension-free vaginal tape-obturator, *TOT* Transobturator tape

*ICIQ-SF* International Consultation on Incontinence Questionnaire –Short Form, *SSI* Sandvik Severity Index, *PGI-I* Patient global impression of improvement, *CST* Cough stress test

^a^ Using Newcastle–Ottawa Scale (score from 0 to 9)

^b^ Using Jadad scale (score from 0 to 5)

### Subjective and objective cure rate

**Fig. 2:**
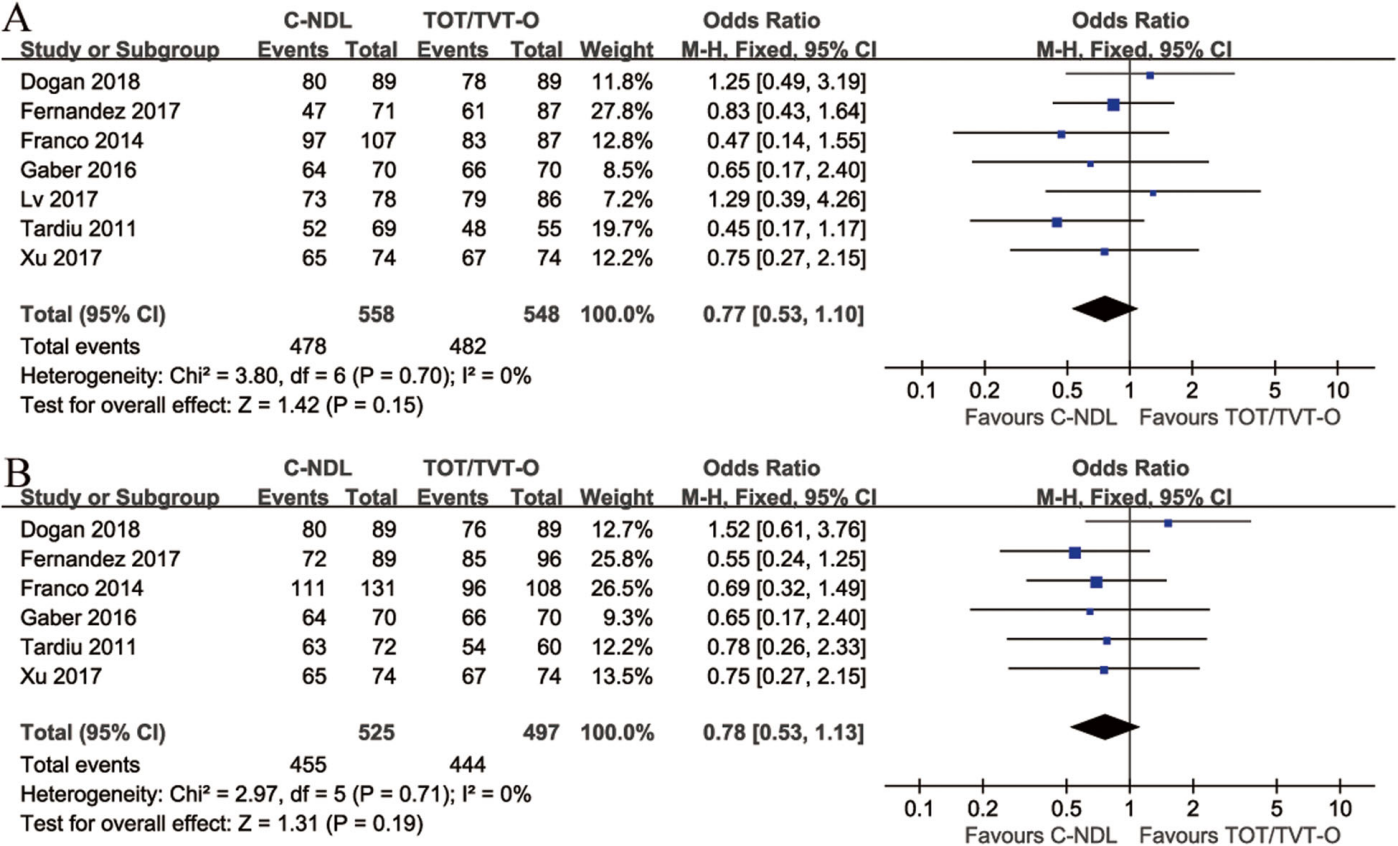
Forest plots and meta-analyses. **a** Subjective cure rate; **b** Objective cure rate [95% CI: 95% confidence intervals, df: degrees of freedom, Fixed: fixed effects model, Random: random effects model, IV: inverse variance, SD: standard deviation]

All 7 studies were included in the forest plot of the subjective cure rate. A fixed-effects model was applied due to the lack of heterogeneity among these trials (I^2^ = 0%). The overall results for the subjective cure rate showed no significant difference between the two groups [OR = 0.77, 95% CI (0.53 to 1.10), *p* = 0.15] (Fig. 2a). For the analysis of the objective cure rate, 6 studies were included. No significant difference was found from the pooled analysis [OR = 0.78, 95% CI (0.53 to 1.13), *p* = 0.19] (Fig. 2b).

### Operation details

#### Operative time

**Fig. 3:**
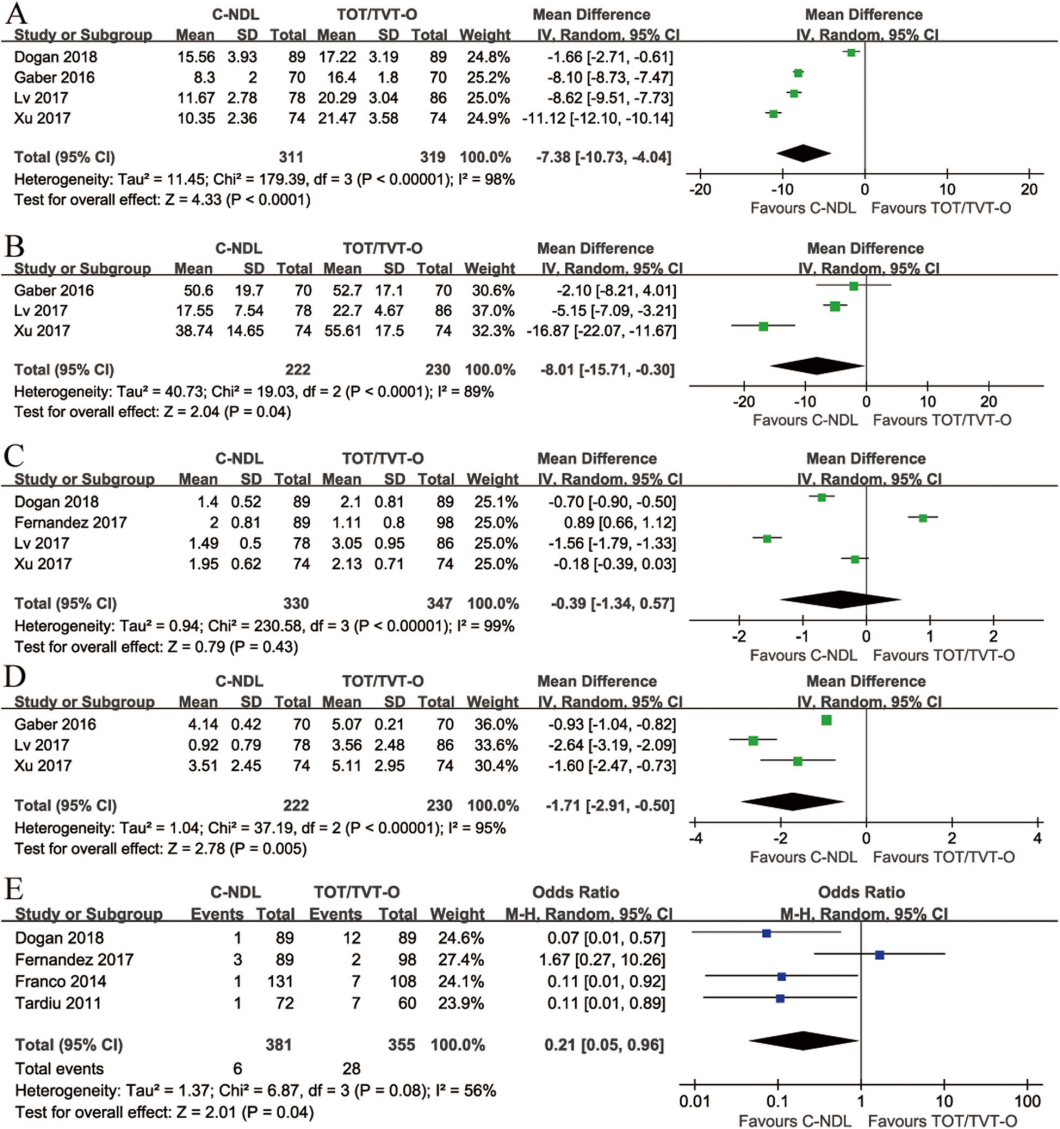
Forest plots and meta-analyses. **a** Operative time; **b** Blood loss; **c** Hospital stay; **d** Postoperative visual analogue scale; **e** Postoperative pain or discomfort [95% CI: 95% confidence intervals, df: degrees of freedom, Fixed: fixed effects model, Random: random effects model, IV: inverse variance, SD: standard deviation]

Four studies measured the intraoperative duration of C-NDL and TOT/TVT-O. We chose the random effects model due to the high heterogeneity (I^2^ = 98%). The analysis results demonstrated that C-NDL slings incurred a shorter operative time than TOT/TVT-O slings [MD = − 7.38, 95% CI (− 10.73 to − 4.04), *p* < 0.0001] (Fig. 3a).

#### Blood loss

Regarding blood loss, the data provided by three studies were analysed by a random effects model with high heterogeneity (I^2^ = 89%). When pooled, the results showed that the C-NDL group had significantly less bleeding [MD = − 8.01, 95% CI (− 15.71 to − 0.30), *p* = 0.04] (Fig. 3b).

#### Hospital stay

Four studies reported data on hospitalization time. A random effects model was used due to the high heterogeneity (I^2^ = 99%). No remarkable difference was found between the two groups [MD = − 0.39, 95% CI (− 1.34 to 0.57), *p* = 0.43] (Fig. 3c).

#### Postoperative visual analogue scale

For the visual analogue scale (VAS), a total of three studies met the inclusion criteria. Due to the high heterogeneity (I^2^ = 95%), we used a random effects model. The combined result showed a significant difference between C-NDL and TOT/TVT-O [MD = − 1.71, 95% CI (− 2.91 to − 0.50), *p* = 0.005] (Fig. 3d).

#### Postoperative pain or discomfort

A total of four studies met the inclusion criteria for this outcome. Due to the high heterogeneity (I^2^ = 56%), a random effects model was applied. The result was statistically significant in favour of C-NDL [OR = 0.21, 95% CI (0.05 to 0.96), *p* = 0.04] (Fig. 3e).

### Adverse events

**Fig. 4:**
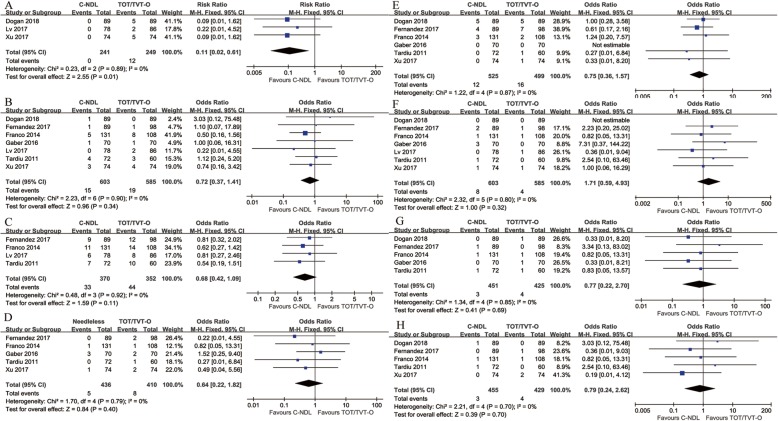
Forest plots and meta-analyses. **a** Postoperative groin pain; **b** Urinary retention; **c** De novo urgency and/or worsening of pre-existing urgency; **d** Difficulty urinating; **e** Vaginal tape erosion; **f** Urinary tract infection; **g** Bladder injury; **h** Hematoma [95% CI: 95% confidence intervals, df: degrees of freedom, Fixed: fixed effects model, Random: random effects model, IV: inverse variance, SD: standard deviation]

Three trials were included in the statistical analysis to detect the incidence rate of postoperative groin pain. The outcome strongly supported that C-NDL slings have a lower incidence rate than TOT/TVT-O slings [RR = 0.11, 95% CI (0.02 to 0.61), *p* = 0.01] **(**Fig. 4a). There were no statistically significant differences between C-NDL and TOT/TVT-O slings in the rate of urinary retention [OR = 0.72, 95% CI (0.37 to 1.41), *p* = 0.34] **(**Fig. 4b), de novo urgency and/or worsening of pre-existing urgency [OR = 0.68, 95% CI (0.42 to 1.09), *p* = 0.11] **(**Fig. 4c), difficulty urinating [OR = 0.64, 95% CI (0.22 to 1.82), *p* = 0.40] **(**Fig. 4d), vaginal tape erosion [OR = 0.75, 95% CI (0.36 to 1.57), *p* = 0.44] **(**Fig. 4e), urinary tract infection [OR = 1.71, 95% CI (0.59 to 4.93), *p* = 0.32] **(**Fig. 4f), bladder injury [OR = 0.77, 95% CI (0.22 to 2.70), *p* = 0.69] **(**Fig. 4g), or haematoma [OR = 0.79, 95% CI (0.24 to 2.62), *p* = 0.70] **(**Fig. 4h).

### Publication bias

**Fig. 5:**
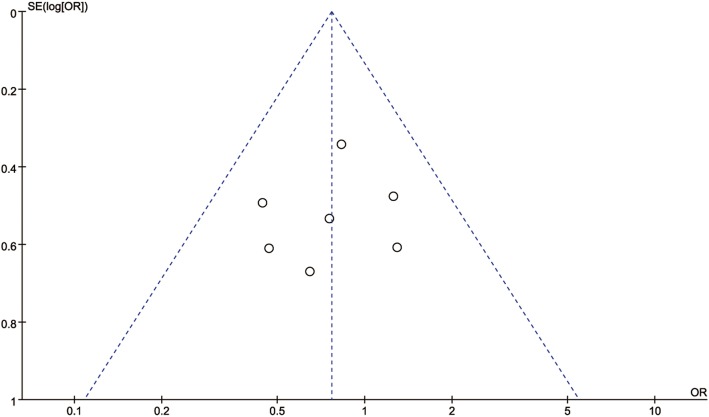
Funnel plot of subjective cure rate for publication bias

To evaluate publication bias, a funnel plot (Fig. 5) was constructed. No obvious asymmetry was shown, revealing that no publication bias existed in our meta-analysis.

## Discussion

With a grade A commendation according to the European Association of Urology (EAU) guidelines, SMUS is widely used to treat SUI [29]. The midurethral slings can be classified into 3 generations throughout their development history. As the first generation, TVT was introduced in 1995 [30] and became the best choice for the surgical treatment of SUI due to its minimal invasiveness and high cure rate. However, as a retropubic sling, the main complications include voiding dysfunction and bladder injuries, which decrease its safety [31, 32]. To avoid potential complications associated with retropubic placement, a mesh inserted through the obturator foramen was created. Delorme introduced the “outside-in” technique (TOT), placing the suburethral tape via a transobturator route in 2001 [33]. Subsequently, the “inside-out” technique (TVT-O) was modified by De Leval [34]. As the second generation, transobturator slings (TOT/TVT-O) achieve similar efficacy and fewer complications, especially regarding the risk of visceral injury [35]. Some studies mainly focused on primary SUI in females without ISD and mixed incontinence were performed to compare the efficacy of the two sling surgeries. The results from a five-year term showed that the subjective and objective cure rates were not significantly different between TVT and TVT-O, and there appeared to be very few complications during follow-up [12]. The meta-analysis performed by Huang et al. [36] showed that TOT can achieve a similar success rate as TVT and requires less operative time and a shorter hospital stay. Few studies reported outcomes with longer term follow-up for more than 5 years. According to the guide for stress urinary incontinence treatment, compared to transobturator slings, slings inserted by the retropubic routes have higher objective patient-reported cure rates at 8 years and lower re-operation rates [37]. In addition, when the tape is placed, the introducer needle passes through the obturator foramen blindly. The obturator nerve and blood vessel will likely be injured during the procedure, which may cause postoperative groin pain and haematoma. According to the existing literature focused on primary SUI in females, transient groin pain occurs in 0.8–7.5% of patients after the placement of transobturator slings [38, 39]. It is worth mentioning that shorter than traditional transobturator slings, TVT-Abbrevo is only 12 cm long, achieves similar efficacy and reduces post-operative groin pain [40, 41]. However, further long-term data and prospective studies are required to clarify its efficacy and safety. To simplify the surgical procedure and decrease the relative complication rate, the third-generation SIMS was introduced. With a shorter mesh and insertion into a single incision, SIMS provides a shorter operative time and fewer adverse effects, particularly regarding postoperative groin pain, vessel and bladder injury [1]. In contrast to other SIMSs, C-NDL has a unique shape. It is made of a polypropylene monofilament mesh with no guide needle and is apparently widened at both ends, which is called the T-pocket. This device aids surrounding tissue ingrowth to provide sufficient support for the urethra [42]. Surgical forceps are placed inside the T-pocket to fold the mesh by opening and closing the forceps. The forceps with the folded mesh are introduced into the paraurethral space and penetrate the internal obturator muscle by controlling the pushing force. The forceps are then opened to extend the T-pocket pocket inside the internal obturator muscles for fixation, closed, and pulled out of the vagina. The same manoeuvre is performed on the contralateral side to complete the sling insertion without twisting the tape. The surgeon can decrease the support by pulling on the two sutures in the middle of the mesh, leaving a space between the mesh and the urethra and allowing a surgical forceps to be interposed between them, thus avoiding any tension on the mesh [24, 25].

The most important indicators for evaluating efficacy are the subjective and objective cure rates. The tension provided by the tape support plays a significant role in efficacy and is associated with the cure rate and necessity for re-surgery. Some studies of SIMS have been performed previously to evaluate the efficacy. One meta-analysis compared the TVT-Secure, Mini-Arc and Ophira together with standard midurethral slings, and the result showed an inferior cure rate for these SIMSs [15]. An animal trial reported that with the highest surface area to counteract extraction, the anchor of the C-NDL has the highest mean immediate extraction forces compared with other SIMSs [43]. Additionally, the surface area in the pelvic floor is almost the same as for the TVT-O, which ensures sufficient firmness [17]. Our meta-analysis, found no significant differences in the comparison of subjective and objective cure rates. However, Fernandez et al. (2017) reported that C-NDL was inferior in a comparison of negative stress test results and patient satisfaction [24]. In contrast to other included studies, they used a non-inferiority test for analysis. However, differences in the statistical tools used could have resulted in bias. In addition, there are no standardized assessment tools to evaluate the success rate. Patients may have different expectations regarding treatment after surgery for SUI, and some postoperative complications may also affect the subjective cure rate. Notably, four studies [17, 24, 25, 27] included patients with pelvic organ prolapse (POP). Because of the pelvic structure, SUI and POP are often comorbid diseases. For these patients, anti-incontinence surgery simultaneous with POP repair was undertaken. There is strong evidence that prolapse surgery combined with incontinence surgery reduces the risk of postoperative SUI [44]. Therefore, it is doubtless that many factors influence the cure rate. Regarding these aspects, more strict inclusion and exclusion criteria and standardized assessment tools are recommended to make the results more comparable. Apart from the investigations of Dogan in 2018 [23] and Franco in 2014 [17], the follow-up time of the included studies was only 1 year. Due to the limitations in follow-up time, only one study [23] (Dogan 2018) with a 2 year follow-up reported the incidence of repeated SUI surgery and revealed no significant differences between groups [C-NDL (1/89): TOT (0/89) *p* > 0.05]. However, longer follow-up is required to determine re-operation rates. Our outcome can only indicate that C-NDL slings have similar short-term efficacy to transobturator slings. Due to the short period of C-NDL use, determination of long-term efficacy will require high quality-RCTs.

Our meta-analysis showed that the patients who underwent C-NDL had a shorter operative time (by 7 min), confirming previously published outcomes [1, 45]. Due to its unique T-pocket, the mesh implant is simpler and more convenient. However, this shorter operative time may not contribute to improving the safety of the operation. Although the pooled results showed a statistically significant reduction in blood loss of 8 ml in the C-NDL group, this difference is not clinically significant. In addition, the pooled results showed that patients receiving C-NDL presented improved postoperative pain and postoperative VAS scores (24 h after operation). In particular, groin pain was greatly improved. These results are consistent with those of Kim’s study [45], which reported that SIMSs are superior with respect to immediate postoperative pain. The difference may be explained by the surgical procedure. Similar to other SIMSs, the C-NDL is inserted through a single vaginal incision and blindly avoids the retropubic space and obturator foramen. The reduction in pain occurrence with C-NDL is related to the fact that there is no incision in the groin area, the sling does not reach the groin, and there is no foreign body sensation. The surgical routine also decreases the risk of blood vessel and nerve injury, consistent with our present analysis. In Baya’s study, after three-year follow-up, there were no cases of groin pain, and only one patient had a mild haematoma [46]. In addition to the cure rate, postoperative pain also plays an important role in patient satisfaction. In a study by Schellart et al., patients with SUI were willing to accept a relatively lower cure rate with a less invasive procedure to avoid postoperative pain [47]. Although these outcomes from our study demonstrated that C-NDL slings cause less pain, some factors should be taken into consideration. Regarding the important outcome of groin pain, only three studies reported the outcome independently; some studies included it in the postoperative pain outcome. The postoperative VAS scores compared the feeling of postoperative pain, which may be influenced by incisions and individual differences. The different anaesthesia protocols among the studies may also contribute heterogeneity to the postoperative VAS results. In addition, the pain evaluation for this surgery is short-term. Lv et al. reported no significant difference between the two groups in VAS at 6 months after surgery, which was associated with the postoperative time [26]. Further studies should pay more attention to groin pain and consider its relative influence. With respect to the length of hospital stay, a previous meta-analysis showed that SIMSs involve a shorter hospitalization time than transobturator slings [48]. However, our results showed no significant differences in hospital stay between the two groups. According to our clinical experience, the length of hospital stay might be associated with the patients’ baseline basic characteristics and with hospital conditions, which are affected by human factors. However, after excluding the study by Fernandez et al., the difference was significant; that study included patients with POP, which may influence the results. In addition, the inpatient stay was fixed at 1 to 2 days, which also indicates that C-NDL slings involve a fast recovery time.

The results of the present meta-analysis, revealed no remarkable difference between the two types of slings with respect to vaginal tape erosion. Some SIMS, such as TVT-Secure and Mini-Arc, have been withdrawn from clinical use due to their nonideal efficacy and high risk of mesh exposure [49, 50]. The C-NDL sling is shorter than the standard sling and thus introduces, less foreign material. Due to the use of monofilament knotless weaving technology, the C-NDL sling is macroporous, with a porosity of 55%; thus, the risk of bacterial retention and reproduction resulting from the dead space caused by knotting after implantation in the human body is reduced [51, 52]. The C-NDL sling also contributes to the reduction in possible complications. In addition, the incidence of urinary retention, de novo urgency and/or worsening of pre-existing urgency, difficulty urinating, urinary tract infection, bladder injury, and haematoma were not significantly different between the groups. Some complications, such as vaginal tape erosion and urinary tract infection, will appear over time. Due to the limitations in follow-up time, long-term safety is still worth observing. Based on the simple operation required, the C-NDL procedure is more suitable for local anaesthesia [42]. Additionally, compared to the traditional sling, the mini-sling is considered lower cost [53, 54]. ElSheemy et al. reported they reduced the cost from US$500 to US$10 by using a surgeon-tailored ordinary polypropylene mesh through the needle-less single-incision technique [55]. More studies are required to verify the cost-effectiveness of the C-NDL procedure in the management of SUI.

There were several limitations to our study. First, the included RCTs did not describe the blinding procedures clearly and in detail, which might have led to conclusion bias. Some potential biases caused by CCTs are inevitable. Moreover, the heterogeneity among these trials was found to be high in terms of several parameters, including operative time, hospital stay, blood loss, and postoperative VAS. The heterogeneity in these parameters can be explained by the differences in outcome definitions, measurements, and standards, such as the time from incision to closure and the overall time spent in the operating room. Haemoglobin levels or gauze was used to estimate blood loss. In addition, the availability of technical equipment and surgical experience also play critical roles in evaluating the effectiveness of the two techniques and could not be assessed in the present review. Another limitation of this study is that some included studies included unselected populations (patients with POP and different severity of SUI), and some patients included in two studies [17, 27] may have come from the same group. Finally, the follow-up times in these included studies were different and not sufficiently long, and the sample sizes and numbers of included studies were small; thus, more high-quality RCTs with long follow-up times are needed to further evaluate the effectiveness and safety of C-NDL. The results of this meta-analysis should be interpreted with caution due to these limitations.

## Conclusion

In summary, C-NDL slings have similar short-term efficacy as TOT/TVT-O slings in curing pure SUI patients without evidence of ISD. Compared with TOT/TVT-O, C-NDL is associated with a shorter operative time (by 7 min) and less blood loss (by 8 ml). In addition, C-NDL is associated with lower postoperative pain compared to TOT/TVT-O. The limitations identified should be taken into consideration when interpreting these results. Further large-volume, well-designed prospective RCTs with extensive follow-up are required to confirm long-term efficacy and safety.

## Data Availability

The datasets used and/or analysed during the current study available from the corresponding author on reasonable request.
